# Sequential segmental analysis

**DOI:** 10.4103/0974-2069.52803

**Published:** 2009

**Authors:** Robert H Anderson, Girish Shirali

**Affiliations:** Department of Pediatric Cardiology, Medical University of South Carolina, Charleston, South Carolina, United States of America

## INTRODUCTION

It might reasonably be thought that those who diagnose and treat patients with congenitally malformed hearts would, by now, have reached consensus concerning the most appropriate way of describing the malformations with which they are confronted. It is certainly the case that nomenclature is far less contentious now than was the case a decade ago. It would be a brave person, nonetheless, who stated that the field of description and categorization was now fully resolved. There are still major differences of opinion as how best to cope with certain topics, such as those patients who have so- called visceral heterotaxy. In this review, we outline a system for description that accounts well for such topics. Indeed, it provides a means of cataloguing and describing all congenital cardiac malformations, even if the combination of lesions has never previously been encountered. In reality, there is no right or wrong way of describing the hearts, simply different ways.[1,2] Even these different ways have been mitigated to considerable extent by the cross-mapping of existing systems.[3] The success of cross-mapping, nonetheless, should not detract from the need to resolve ongoing differences according to the nature of the abnormal anatomy as it is observed. In this review, we provide such accounts of the phenotypic features of the so-called cardiac segments. We show how this approach then provides the template for categorising the arrangements in all patients with congenitally malformed hearts.

## THE BASIC APPROACH TO CATEGORISATION

In terms of its basic make-up, the heart has three building blocks, namely the atriums, the ventricular mass, and the arterial trunks. The first systems of categorization based on recognition of the limited potential for variation in each of these cardiac segments were developed independently in the 1960s by two groups: one based in the United States of America, and led by Richard Van Praagh,[1] and the other, from Mexico City, headed by Maria Victoria de la Cruz.[4] Both of these systems concentrated on the different topological arrangements of the components within each cardiac segment. At that time, these approaches were understandable, since it was often difficult with the diagnostic techniques then available precisely to determine how the adjacent structures were linked together.

All of this changed with the advent of cross-sectional echocardiography. Since the mid 1970s, it has been possible with precision to determine how atriums are, or are not, joined to ventricles, and similarly to establish the precise morphology found at the ventriculo-arterial junctions. Thus, the system with which we have been involved was produced concomitantly with the development of echocardiography, with attention concentrated on the potential variations to be found across the atrioventricular and ventriculo-arterial junctions. The system was called, and is still called, sequential segmental analysis.[2,5–7] It should not be thought that the topology of the segments themselves is ignored when making such analysis. Junctional connections cannot be established without initial knowledge of segmental topology.

During its evolution, the system has followed some basic and simple rules. From the outset, categories have been based on recognizable anatomical features, eschewing speculative embryological assumptions. Emphasis is placed on the morphology of the cardiac components, the way they are joined or not joined together, and the relations between them, as three different facets of the cardiac make-up. Any system that separates these features one from the other, does not use one to determine another, and describes them with mutually exclusive terms, must perforce be unambiguous. The clarity of the system then depends upon its design. Some systems opt for brevity, with formidable codifications constructed to achieve this aim.[8] But clarity is surely more important than brevity? We do not shy, therefore, from using words to replace symbols, even if this requires several words. Wherever possible, we strive to use words that are as meaningful in their systematic role as in their everyday usage. In the desire to achieve optimal clarity, changes have been made in our descriptions over the years. We make no apologies for these changes, since their formulation, in response to valid criticisms, has eradicated aspects of the system that were initially illogical. Having expunged these aspects, it is our belief that the system now advocated is entirely logical, and is also simple.

## THE ESSENCE OF SEQUENTIAL SEGMENTAL ANALYSIS

The system depends first upon the establishment of the arrangement of the atrial chambers. Attention is then concentrated on the anatomical nature of the junctions between the atrial myocardium and the ventricular myocardial mass. This feature, described as a type of connection, is separate from the additional feature of the morphology of the valve or valves that guard the junctions [Flow diagram]. The normally constructed heart possesses 2 atrioventricular junctions, usually with each junction guarded by its own atrioventricular valve. On occasion, the two junctions can be guarded by a common valve. In order to achieve such analysis of the junctions, it is essential first to have determined the structure, topology, and relationships of the chambers within the ventricular mass. Having dealt with the atrioventricular junctions, the ventriculo-arterial junctions are analysed according to how the arterial trunks are joined to the ventricular mass, along with the morphology of the arterial valves guarding their junctions. Separate attention is directed to the morphology of the outflow tracts, and to the relationships of the arterial trunks. Once segmental connections have been established, and note taken of appropriate relationships, a catalogue is then made of all associated cardiac, and where pertinent, non-cardiac, malformations. Included in this final category are such features as the location of the heart, the orientation of its apex, and the arrangement of the other thoracic and abdominal organs.

**Flow diagram:**
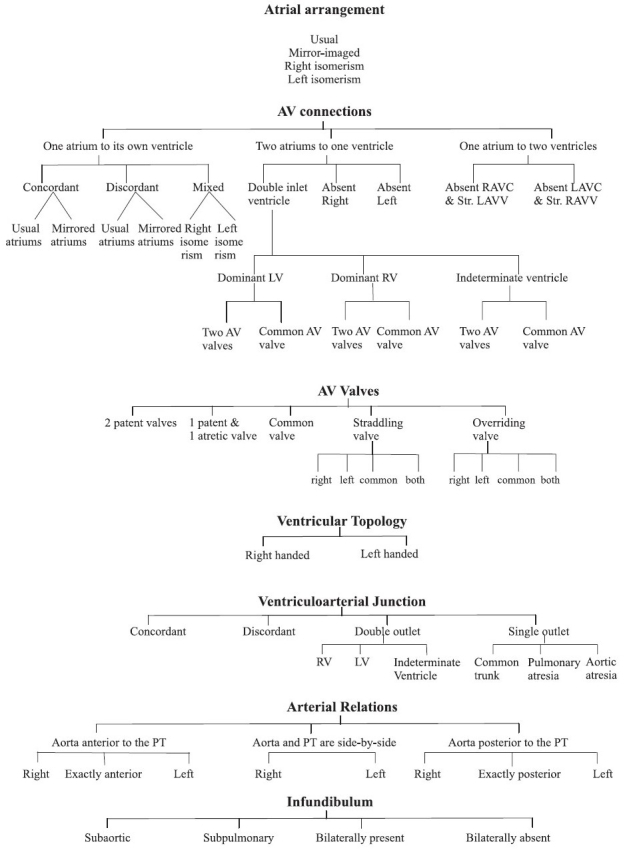
Variable features in sequential segmental analysis

**Figure 1:**
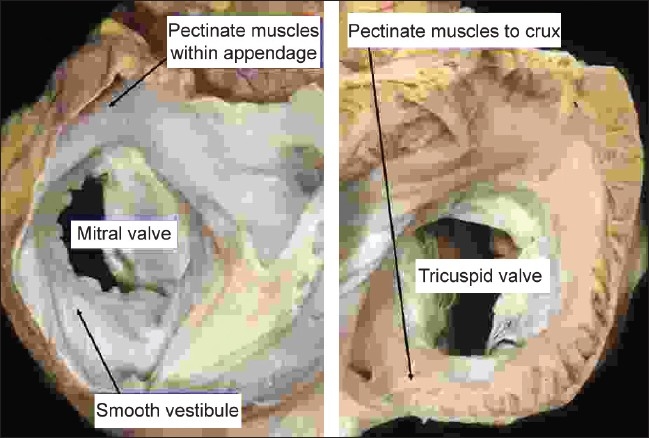
The images show the short axis views of the left (left hand panel) and right (right hand panel) atrioventricular junctions of the normal heart as seen from above, having opened the atriums with a cut parallel to the atrioventricular junctions. They show how, in the morphologically right atrium (right hand panel), the pectinate muscles within the the appendage extend all round the vestibule of the tricuspid valve. In the morphologically left atrium, in contrast (left hand panel), the pectinate muscles are confined within the tubular appendage, so that the inferior wall of the atrium is smooth

Implicit in the system is the ability to distinguish the morphology of the individual atriums and ventricles, along with the pattern of branching of the arterial trunks taking origin from the ventricles. This is not as straightforward as it may seem, since often, in congenitally malformed hearts, the chambers or arterial trunks may lack some of the morphological features that most obviously characterize them in the normal heart. For example, the most obvious feature of the morphologically left atrium in the normal heart is the connection to it of the pulmonary veins. Hearts with totally anomalous pulmonary venous connection, for example, lack such a feature. In spite of this lack of pulmonary venous connection, it is almost always still possible to identify the left atrium. Considerations of this type prompted the concept now used for recognition of the cardiac chambers and great arteries. Called the morphological method,[9] the principle states that structures should be recognized in terms of their own intrinsic morphology. A part of the heart that is itself variable, therefore, should not be defined on the basis of another variable structure. When this concept is applied to the atrial chambers, the connections of the great veins are obviously disqualified as markers of morphological rightness or leftness since, as discussed above, the veins do not always connect to their anticipated atriums. The morphology of the septum is also of little help when the septum itself is absent, as occurs in the setting of a common atrium. Similarly, the atrial vestibule is ruled out as a marker, since it provides no distinguishing features for the right as opposed to the left atrium. There remains one component of the atrial chambers that, in our experience, has been almost universally present and which, on the basis of the morphology of its junction with the remainder of the chambers, has enabled us always to distinguish between morphologically right and left atriums. This is the appendage. The morphologically right appendage has the shape of a blunt triangle, and joins over a broad junction with the remainder of the atrium. Its most significant feature is the pectinate muscles within the appendage that extend all round the parietal atrioventricular junction [Figure 1 – right hand panel].

The morphologically left appendage, in contrast, is much narrower and tubular. It has a narrow junction with the remainder of the atrium. The pectinate muscles are confined within the appendage, with the posterior aspect of the morphologically left vestibule, also containing the coronary sinus, being smooth walled as it merges with the body of the atrium [Figure 1 – left hand panel].

**Figure 2:**
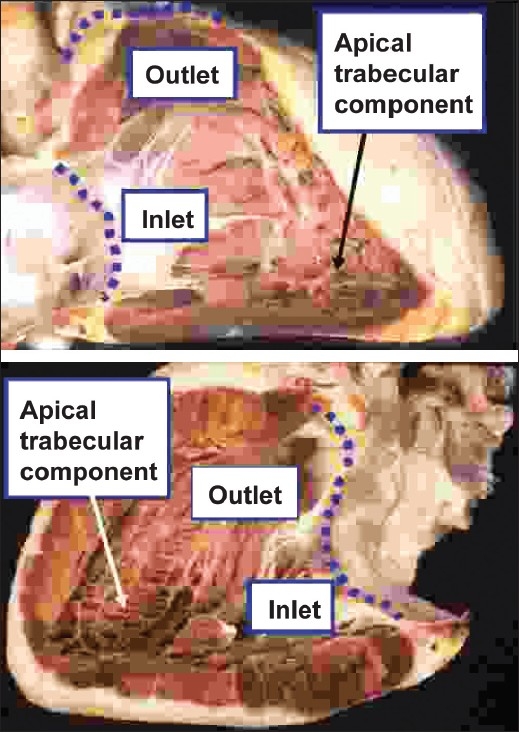
The upper panel shows the morphologically right ventricle, which extends from the atrioventricular to the ventriculo-arterial junctions (dotted red lines), with the anterior wall removed to show its three component parts. The coarse apical trabeculations are the most constant of these features. The lower panel shows the comparable three component parts of the morphologically left ventricle of the same heart, revealed by removing its posterior wall. This ventricle also extends from the atrioventricular to the ventriculo-arterial junctions (dotted purple lines). Its fine apical trabeculations are its most constant feature, and distinguish it from the morphologically right ventricle

**Figure 3:**
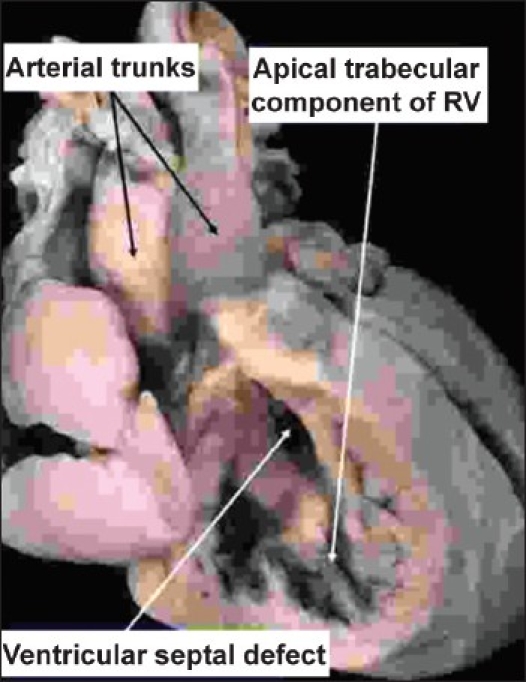
In the heart illustrated, there is double inlet to, and double outlet from a dominant left ventricle. The arterial trunks are seen arising in parallel fashion from the left ventricle, with the aorta anterior and to the left. On the anterior and right-sided shoulder of the dominant left ventricle, however, there is still a second chamber to be seen, fed through a ventricular septal defect. This chamber is the apical trabecular component of the right ventricle (RV), identified because of its coarse trabeculations

The morphological method also shows its value when applied to the ventricular mass, which extends from the atrioventricular to the ventriculo-arterial junctions. Within the ventricular mass as thus defined, there are almost always two ventricles. Description of ventricles, no matter how malformed they may be, is facilitated if they are analysed as possessing three components. These are, first, the inlet, extending from the atrioventricular junction to the distal attachment of the atrioventricular valvar tension apparatus. The second part is the apical trabecular component. The third is the outlet component, supporting the leaflets of the arterial valve. Of these three components, it is the apical trabecular component that is most universally present in normal as well as in malformed and incomplete ventricles, and which most readily differentiates morphologically right from left ventricles [Figure 2]. This is the case even when the apical components exist as incomplete ventricles, which sometimes lack both their inlet and outlet components [Figure 3].

In order fully to describe any ventricle, account must also be taken of its size. It is then necessary further to describe the way that the two ventricles themselves are related within the ventricular mass. This feature is described in terms of ventricular topology, since two basic patterns are found that cannot be changed without physically taking apart the ventricular components and reassembling them. The two patterns are mirror images of each other. They can be conceptualized in terms of the way that, figuratively speaking, the palmar surface of the hands can be placed upon the septal surface of the morphologically right ventricle. In the morphologically right ventricle of the normal heart, irrespective of its position in space, only the palmar surface of the right hand can be placed on the septal surface such that the thumb occupies the inlet and the fingers fit into the outlet. The palmar surface of the left hand then fits in comparable fashion within the morphologically left ventricle, but it is the right hand that is taken as the arbiter for the purposes of categorization. The usual pattern, therefore, can be described as right hand ventricular topology. The other pattern, the mirror image of the right hand prototype, is then described as left hand ventricular topology. In this left hand pattern, seen typically in the mirror-imaged normal heart, or in the variant of congenitally corrected transposition found with usual atrial arrangement, it is the palmar surface of the left hand that fits on the septal surface of the morphologically right ventricle with the thumb in the inlet and the fingers in the outlet. These two topological patterns can always be distinguished irrespective of the location occupied in space by the ventricular mass itself. Component make-up, trabecular pattern, topology, and size are independent features of the ventricles. On occasion, all may need separate description in order to remove any potential for confusion.

**Figure 4:**
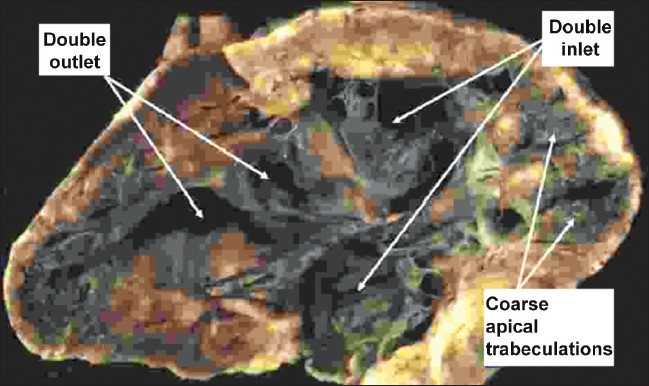
The heart is opened in clamshell fashion to show that both atrioventricular valves enter the same ventricular chamber, which also gives rise to both outflow tracts. We were unable to find a second ventricular chamber. The exceedingly coarse apical trabeculations, and the absence of the second chamber, identify this heart as having a solitary ventricle of indeterminate morphology

Only rarely will hearts be found with a solitary ventricle. Sometimes this may be because a right or left ventricle is so small that it cannot be recognized with usual clinical investigatory techniques. There is, nonetheless, a third pattern of apical ventricular morphology that is found in hearts possessing a truly single ventricle. This is when the apical component is of neither right or left type, but is very coarsely trabeculated, and crossed by multiple large muscle bundles. Such a solitary ventricle has an indeterminate apical morphology [Figure 4]. Analysis of ventricles on the basis of their apical trabeculations precludes the need to use illogically the terms single ventricle, or univentricular heart, for description of hearts having one big and one small ventricle.[10,11] Any attempt to disqualify such chambers from ventricular state must lead to a system of nomenclature that is artificial and anatomically inaccurate. Only hearts with a truly solitary ventricle should be described as being anatomically univentricular, albeit that the connections across the atrioventricular junctions can be univentricular in many more hearts.

When determining the morphology of the arterial trunks, there are no intrinsic features that enable an aorta to be distinguished from a pulmonary trunk, or from a common or solitary arterial trunk. The branching pattern of the trunks themselves, nonetheless, is always sufficiently characteristic to permit these distinctions. The aorta gives rise to at least one coronary artery and the bulk of the systemic arteries. The pulmonary trunk gives rise directly to both, or one or other, of the pulmonary arteries. A common trunk supplies directly the coronary, systemic and pulmonary arteries. A solitary arterial trunk exists in the absence of the proximal portion of the pulmonary trunk. In such circumstances, it is impossible to state with certainty whether the persisting trunk is common or aortic. Even in the rare cases that have transgressed one of these rules, examination of the overall branching pattern has always permitted us to distinguish the nature of the arterial trunk.

## ATRIAL ARRANGEMENT

**Figure 5:**
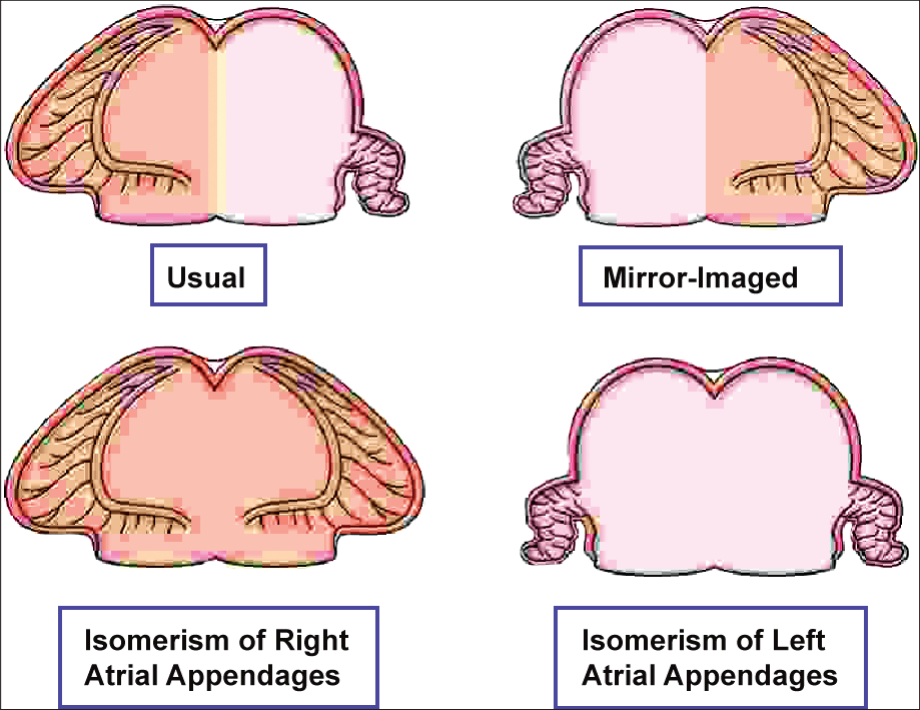
The cartoon shows how, when analysed on the basis of the extent of the pectinate muscles relative to the atrioventricular vestibules, there are only four possible ways for arrangement of two atrial appendages

The cornerstone of any system of sequential analysis must be accurate establishment of the arrangement of the atrial chambers, since this is the starting point for subsequent analysis. When this arrangement is assessed on the basis of the morphology of the junction of the appendages with the rest of the atriums, then since all hearts have two atrial appendages, each of which can only be of morphologically right or left type, there are only four possible patterns [Figure 5]. The most common is the usual arrangement, also called situs solitus, in which the morphologically right appendage is right-sided, and the morphologically left appendage is left-sided. The second arrangement, very rare, is the mirror image of the usual. It is often called situs inversus, even though the atrial chambers are not upside down. In these two arrangements, the appendages are lateralized, with the morphologically right appendage being to one side, and the morphologically left appendage to the other. The two other arrangements do not show such lateralization. Instead, there is isomerism of the atrial appendages. In these patterns, the two appendages are mirror images of each other, with morphological characteristics at their junctions with the rest of the atriums on both sides of either right type or left type.

In the ideal world, the arrangement of the appendages will be recognized by direct examination of the extent of the pectinate muscles round the vestibules [Figure 1]. In skilled hands, particularly when using three-dimensional technology, and noting the presence of the coronary sinus within the morphologically left junction, this feature should now be recognizable using cross-sectional echocardiography, particularly from the transoesophageal window. In most clinical situations, it is rarely necessary to rely only on direct identification. This is because, almost always, the morphology of the appendages is in harmony with the arrangements of the thoracic and abdominal organs. In patients with lateralized arrangements, in other words with the usual and mirror imaged patterns, it is exceedingly rare for there to be disharmony between the location of the organs. When the appendages are isomeric, in contrast, then usually the abdominal organs are typically jumbled-up. Even when there is such abdominal heterotaxy, the lungs and bronchial tree are almost always symmetrical, and it is rare for the bronchial arrangement to show disharmony with the morphology of the appendages. The presence of isomerism, therefore, can almost always be inferred from the bronchial anatomy. The morphologically left bronchus is long, and it branches only after it has been crossed by its accompanying pulmonary artery, making the bronchus hyparterial. In contrast, the morphologically right bronchus is short, and is crossed by its pulmonary artery only after it has branched, giving an eparterial pattern of branching. The four patterns of bronchial branching are then almost always in harmony with the arrangement of the atrial appendages. Similar inferences to those provided from bronchial arrangement can also usually be obtained non-invasively by using cross-sectional ultrasonography to image the abdominal great vessels. These vessels bear a distinct relation to each other, and to the spine, which generally reflects bodily arrangement, although not as accurately as does bronchial anatomy. The vessels can be distinguished ultrasonically according to their pattern of pulsation. When the atriums are lateralized, then almost without exception the inferior caval vein and aorta lie to opposite sides of the spine, with the caval vein on the side of the morphologically right appendage. When there is isomerism, then the great vessels usually lie to the same side of the spine, with the caval vein in anterior position in those with isomerism of the right atrial appendages, and posterior, or with the azygos vein posterior, in those having isomerism of the left atrial appendages.

Generally speaking, isomerism of the right atrial appendages is associated with absence of the spleen, while isomerism of the left atrial appendages is associated with multiple spleens. Patients with isomerism of the atrial appendages, therefore, are frequently grouped together, from the cardiac standpoint, under the banner of the splenic syndromes. This approach is much less accurate than describing the syndromes directly in terms of isomerism of the atrial appendages, since the correlation between isomerism of the right atrial appendages and absence of the spleen, and between isomerism of the left atrial appendages and multiple spleens, is far from perfect.[12] Isomerism of the right and left appendages, in contrast, describes what is there, and additionally serves to concentrate attention upon the heart.

## THE ATRIOVENTRICULAR JUNCTIONS

In the normal heart, the atrial myocardium is contiguous with the ventricular mass around the orifices of the mitral and tricuspid valves. Other than at the site of the penetration of the bundle of His, electrical insulation is provided at these junctions by the fibrofatty atrioventricular grooves. In order to analyse accurately the morphology of the atrioventricular junctions in abnormal hearts, it is first necessary to know the atrial arrangement. Equally, it is necessary to know the morphology of the ventricular mass so as to establish which atrium is connected to which ventricle. With this information to hand, it is then possible to define the specific patterns of union or non-union across the junctions, and to determine the morphology of the valves guarding the atrioventricular junctions. In hearts with complex malformations, it is also necessary, on occasion, to describe the precise topology of the ventricular mass, and to specify the relationships of the ventricles themselves.

## PATTERNS OF UNION OR NON-UNION OF THE ATRIAL AND VENTRICULAR CHAMBERS

These patterns depend on the way that the myocardium of the atrial chambers is joined to the ventricular myocardium around the entirety of the atrioventricular junctions. The cavities of the atrial chambers are potentially connected to the underlying ventricular cavities via the atrioventricular orifices. In every heart, since there are two atrial chambers, there is the possibility for two atrioventricular connections, which will be right-sided and left-sided. This is the case irrespective of whether the junctions themselves are guarded by two valves or a common valve. One of the junctions may be blocked by an imperforate valvar membrane, but this does not alter the fact that, in such a setting, there are still two potential atrioventricular connections. In some hearts, in contrast, this possibility is not fulfilled. This is because one of the connections is absent. The atrial myocardium on that side then has no direct connection with the underlying ventricular myocardium, being separated from the ventricular mass by much more extensive formation than normal of the fibrofatty tissue of the atrioventricular groove. This arrangement is the most common pattern producing atrioventricular valvar atresia.

When atrioventricular connections are defined in this fashion, all hearts fit into one of three groups. In the first group, by far the most common, the cavity of each atrial chamber is in actual or potential, and separate, connection with the cavity of an underlying ventricle. The feature of the second group is that only one of the ventricles, if indeed two are present, is in communication with the atrial cavities. There is then an even rarer third group. This is seen when one atrioventricular connection is absent, and the solitary atrioventricular junction, via a straddling valve, is connected to two ventricles. This arrangement is uniatrial but biventricular.

**Figure 6:**
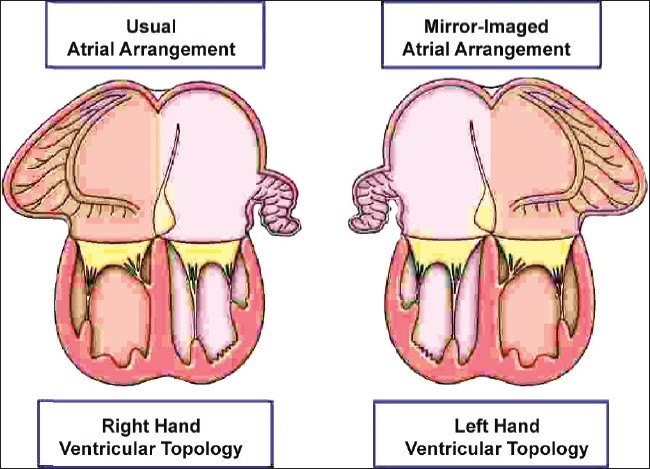
The cartoon shows how concordant atrioventricular connections can exist is usual and mirror-imaged patterns. Almost without exception, atriums with usually arranged appendages are joined to a ventricular mass with right hand topology, whilst atriums with mirror-imaged appendages are joined to a ventricular mass with left hand topology. Except when these associations are not present, it is not necessary also to state the topology of the ventricles

**Figure 7:**
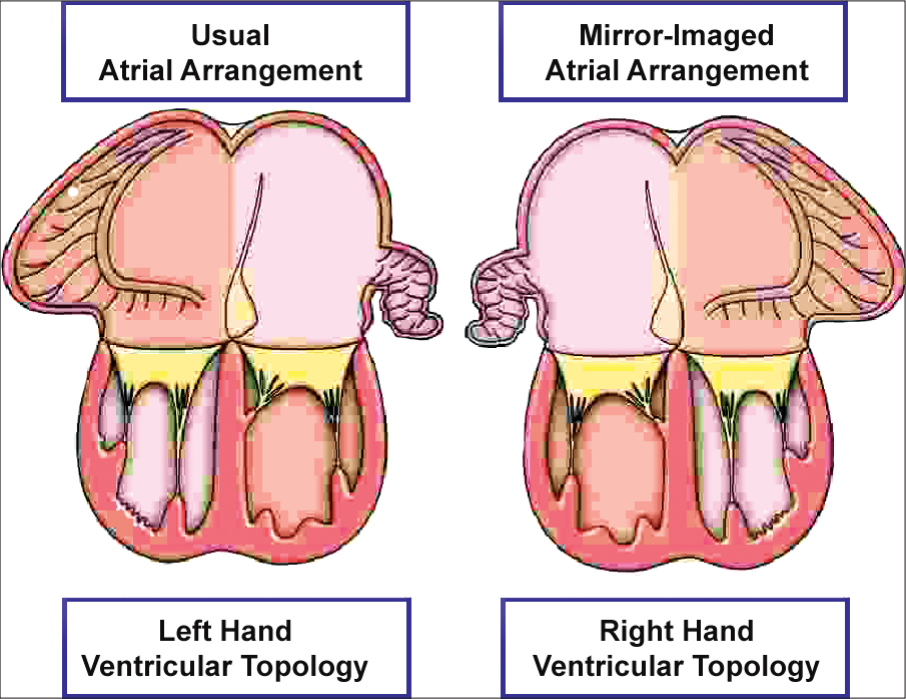
In this cartoon, the arrangements are shown that produce, almost without exception, discordant atrioventricular connections

There are 3 possible arrangements in hearts with each atrium joined to its own ventricle. These depend on the morphology of the chambers connected together. The first pattern is seen when the atriums are joined to morphologically appropriate ventricles, irrespective of the topology or relationship of the ventricles, or of the morphology of the valves guarding the junctions. This arrangement produces concordant atrioventricular connections, and exists with either usually arranged atrial appendages, or in the mirror-imaged arrangement [Figure 6]. The second arrangement is the reverse of the first. It is again independent of relationships or valvar morphology. It produces discordant atrioventricular connections. When it is the atrial appendages that are mirror-imaged in patients with discordant atrioventricular connections, the ventricles are typically in their expected pattern, showing right hand topology [Figure 7].

These first two arrangements are found when the atrial appendages are lateralized. The other biventricular atrioventricular arrangement is found in hearts with isomeric appendages, be they of right or left morphology. This third arrangement cannot accurately be described in terms of concordant or discordant connections. Rather, it is a discrete biventricular pattern in its own right, which is mixed. It, too, is independent of ventricular relationships and atrioventricular valvar morphologies, and requires specification of ventricular topology to make the description complete.

**Figure 8:**
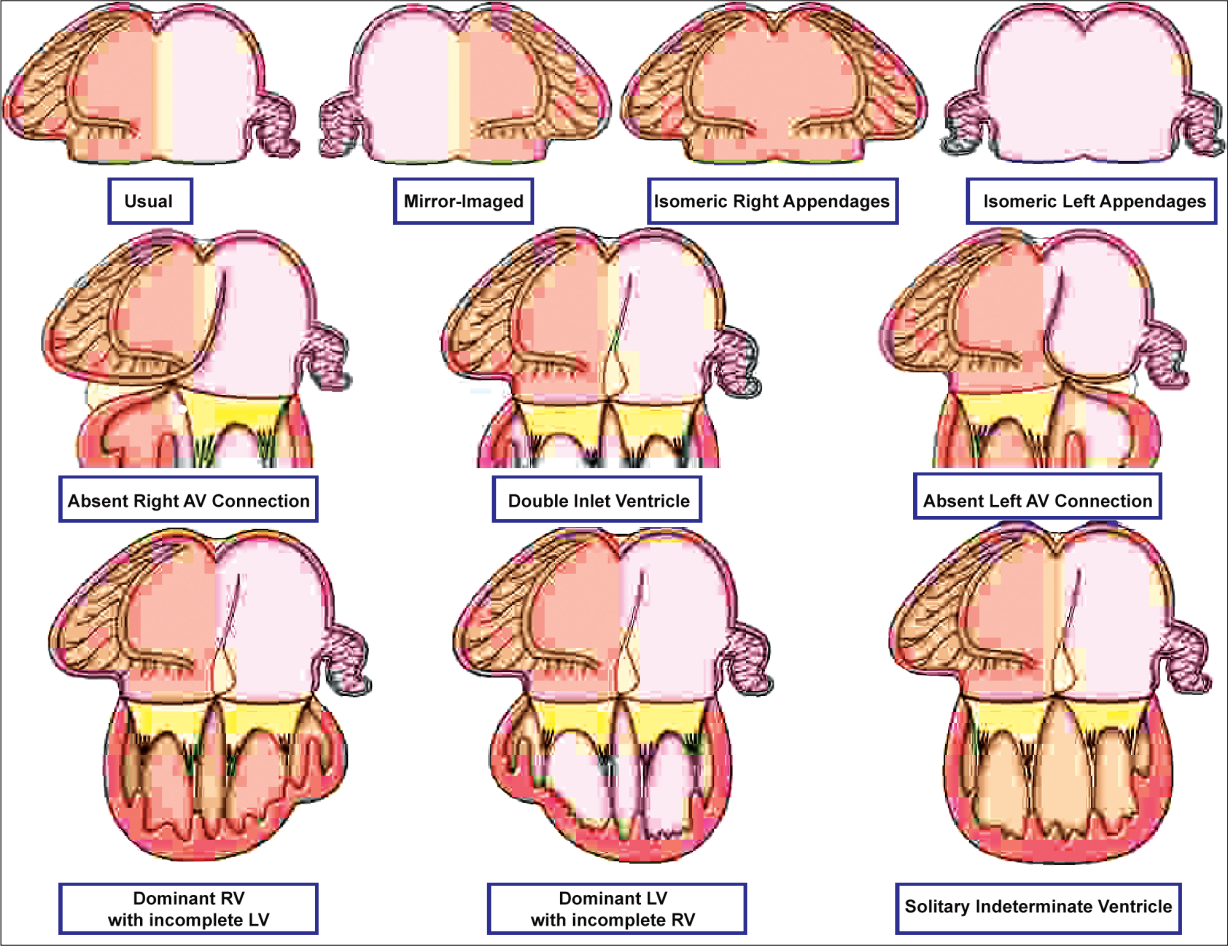
The cartoon shows some of the potential univentricular atrioventricular connections. In reality, these can exist with any arrangement of the atrial appendages (upper row), with double inlet, absent right, or absent left atrioventricular connections (middle row), and with dominant left or right ventricles, or solitary and indeterminate ventricle (bottom row). The possibilities are illustrated with usual arrangement of the atrial appendages simply for convenience, while the examples in the bottom row are shown only for double inlet ventricle. The same variation in ventricular morphology exists for hearts with absence of one atrioventricular connection. There is further variability with regard to the position of the incomplete ventricle, and with ventriculo-arterial connections, and so on. These hearts, therefore, exemplify the need for full sequential segmental analysis and description

There are also 3 possible junctional arrangements that produce univentricular atrioventricular connections [Figure 8]. The first is when the cavities of right-and left-sided atrial chambers are connected directly to the same ventricle. This is called double inlet atrioventricular connection, irrespective of whether the right- and left-sided atrioventricular junctions are guarded by two atrioventricular valves or a common valve. The other two arrangements exist when either the right-sided or left-sided atrioventricular connection is absent. The patterns producing univentricular atrioventricular connections are not only independent of ventricular relationships and valvar morphology, but also independent of atrial and ventricular morphologies. Thus, double-inlet, absent right-sided, or absent left-sided atrioventricular connections can be found with usually arranged, mirror-imaged or isomeric atrial appendages, and with the atriums connected to a dominant right ventricle, a dominant left ventricle, or to a morphologically indeterminate ventricle [Figure 8]. Ventricular morphology must always, therefore, be described separately in those hearts in which the atrial chambers are joined to only one ventricle. Although, in these hearts, only one ventricle is joined to the atriums, in most of them there is a second ventricle present. This second ventricle, of necessity incomplete, will be of complementary trabecular pattern to the dominant ventricle. Most frequently, the dominant ventricle is a left ventricle, and the incomplete ventricle possesses right ventricular apical trabeculations. More rarely, the dominant ventricle is morphologically right, with the incomplete ventricle being morphologically left. Even more rarely, hearts will be found with a solitary ventricular chamber of indeterminate morphology [Figure 4]. In clinical practice, seemingly solitary left or right ventricles may be encountered when the complementary incomplete ventricle is too small to be demonstrated.

## ARRANGEMENTS OF THE ATRIOVENTRICULAR VALVES

Describing the fashion in which the atriums are joined to the ventricles across the atrioventricular junctions accounts only for the way in which the atrial musculature inserts into the base of the ventricular mass. The morphology of the valves guarding the overall atrioventricular junctional area is independent of this feature, within the constraints imposed by the pattern of the junctions itself. When the cavities of both atriums are joined directly to the ventricular mass, the right- and left-sided atrioventricular junctions may be guarded by two patent valves, by one patent valve and one imperforate valve, by a common valve, or by straddling and overriding valves. These arrangements of the valves can be found with concordant, discordant, biventricular and mixed, or double inlet types of connection. Either the right- or left-sided valve may be imperforate, producing atresia but in the setting of a potential as opposed to an absent atrioventricular connection. A common valve guards both right- and left-sided atrioventricular junctions, irrespective of its morphology. A valve straddles when its tension apparatus is attached to both sides of a septum within the ventricular mass. It overrides when the atrioventricular junction is connected to ventricles on both sides of a septal structure. A right-sided valve, a left-sided valve, or a common valve can straddle, can override, or can straddle and override. Very rarely, both right- and left-sided valves may straddle and/or override in the same heart. When one atrioventricular connection is absent, then the possible modes of connection are greatly reduced. This is because the valve, of necessity, is solitary. The single valve is usually committed in its entirety to one ventricle. More rarely, it may straddle, override, or straddle and override. These latter patterns produce the extremely rare group of uniatrial but biventricular connections.

A valve that overrides has an additional influence on description, since the degree of commitment of the overriding atrioventricular junction to the ventricles determines the precise fashion in which the atriums and ventricles are joined together. Hearts with two valves, in which one valve is overriding, are anatomically intermediate between those with, on the one hand, biventricular and, on the other hand, univentricular atrioventricular connections. When most of an overriding junction is connected to a ventricle that is also joined to the other atrium, the pattern is effectively double inlet. If the overriding junction is connected mostly to a ventricle not itself joined to the other atrium, then the pattern is more akin to concordant, discordant, or mixed connections, and is categorized in one of these fashions.

When describing atrioventricular valves, the adjectives mitral and tricuspid are strictly accurate only in hearts with biventricular atrioventricular connections having separate junctions, each guarded by its own valve. In this context, the tricuspid valve is always found in the morphologically right ventricle, and the mitral valve in the morphologically left ventricle. In hearts with biventricular atrioventricular connections but with a common junction, in contrast, it is incorrect to consider the common valve as having mitral and tricuspid components, even when it is divided into right and left components. These right- and left-sided components, particularly on the left side, bear scant resemblance to the normal atrioventricular valves. In hearts with double inlet, the two valves are again better considered as right- and left-sided valves rather than as mitral or tricuspid, as is the case when one connection is absent. Valves can always accurately be described as being right or left sided.

## VENTRICULAR TOPOLOGY AND RELATIONSHIPS

Even in the normal heart, the ventricular spatial relationships are complex. The inlet portions are more or less to the right and left, with the inferior part of the muscular ventricular septum lying in an approximately sagittal plane. The outlet portions are more or less anteroposteriorly related, with the septum between them in an approximately frontal plane. The trabecular portions extend between these two components, with the trabecular muscular septum spiralling between the inlet and outlet components. It is understandable that there is a desire to have a shorthand term to describe such complex spatial arrangements. We use the concept of ventricular topology for this purpose. When applying this concept, the morphologically right ventricle is considered in the way the palmar surfaces of the hands can be applied to its septal surface such that the thumb is in the inlet, and the fingers in the outlet, with the wrist occupying the apical trabecular component. This produces right hand and left hand patterns. In persons with usually arranged atriums and discordant atrioventricular connections, the ventricular mass almost always shows left-handed topological pattern, whereas right-handed ventricular topology is usually found with the combination of mirror-imaged atriums and discordant atrioventricular connections. Although these combinations are almost always present, exceptions can occur. When noting such unexpected ventricular relationships as a feature independent of the topology, we account for right-left, anterior-posterior and superior-inferior coordinates. And, should it be necessary, we describe the position of the three ventricular components separately, and relative to each other.

In hearts with disharmonious arrangements in the setting of usual atrial arrangement and discordant atrioventricular connections, the distal parts of the ventricles are usually rotated so that the morphologically right ventricular trabecular and outlet components are to the right of their morphologically left ventricular counterparts, giving the impression of normal relationships. In such criss-cross hearts seen with usual atrial arrangement and concordant atrioventricular connections, the ventricular rotation gives a spurious impression of left-handed topology. In cases with extreme rotation, the inlet of the morphologically right ventricle may also be right sided in association with discordant atrioventricular connections. Provided relationships are described accurately, and separately, from the connections and the ventricular topology, then none of these unusual and apparently complex hearts will be difficult either to diagnose or to categorize. In addition to these problematic criss-cross hearts, we have already discussed how description of ventricular topology is essential when accounting for the combination of isomeric appendages with biventricular mixed atrioventricular connections. This is because, in this situation, the same terms would appropriately be used to describe the heart in which the left-sided atrium was connected to a morphologically right ventricle as well as the heart in which the left-sided atrium was connected to a morphologically left ventricle. The arrangements are differentiated simply by describing also the ventricular topology.

Both the position and the relationships of incomplete ventricles need to be described in hearts with univentricular atrioventricular connections. Here, the relationships are independent of both the connections and the ventricular morphology. While, usually, the incomplete right ventricle is anterior and right sided in classical tricuspid atresia, it can be anterior and left sided without in any way altering the clinical presentation and haemodynamic findings. Similarly, in hearts with double inlet ventricle, the position of the incomplete ventricle plays only a minor role in determining the clinical presentation. When we describe the position of incomplete ventricles, therefore, we simply account for their location relative to the dominant ventricle, taking note again when necessary of right-left, anterior-posterior and superior-inferior coordinates. On occasion, it may also be advantageous to describe separately the position of trabecular and outlet components of an incomplete ventricle.

## THE VENTRICULO-ARTERIAL JUNCTIONS

Most polemics concerning the ventriculo-arterial junctions devolved upon the failure to distinguish between the way the arterial trunks arose from the ventricular mass as opposed to their relations to each other, along with undue emphasis on the nature of the infundibulums supporting their arterial valves. When these features are described independently, following the precepts of the morphological method, then all potential for disagreement is removed.

## ORIGIN OF THE ARTERIAL TRUNKS FROM THE VENTRICULAR MASS

As with analysis of the atrioventricular junctions, it is necessary to account separately for the way the arteries take origin, and the nature of the valves guarding the ventriculo-arterial junctions. There are four possible types of origin. Concordant ventriculo-arterial connections exist when the aorta arises from a morphologically left ventricle, and the pulmonary trunk from a morphologically right ventricle, be the ventricles complete or incomplete. The arrangement where the aorta arises from a morphologically right ventricle or its rudiment, and the pulmonary trunk from a morphologically left ventricle or its rudiment, produces discordant ventriculo-arterial connections. Double outlet connection is found when both arteries are connected to the same ventricle, which may be of right ventricular, left ventricular or indeterminate ventricular pattern. As with atrioventricular valves, overriding arterial valves are assigned to the ventricle supporting the greater parts of their circumference. The fourth ventriculo-arterial connection is single outlet from the heart. This may take one of four forms. A common trunk exists when both ventricles are connected via a common arterial valve to one trunk that gives rise directly to the coronary arteries, at least one pulmonary artery, and the majority of the systemic circulation. A solitary arterial trunk exists when it is not possible to identify any remnant of an atretic pulmonary trunk within the pericardial cavity. The other forms of single outlet are single pulmonary trunk with aortic atresia, or single aortic trunk with pulmonary atresia. These latter two categories describe only those arrangements in which, using clinical tecniques, it is not possible to establish the precise connection of the atretic arterial trunk to a ventricular cavity. If its ventricular origin can be established, but is found to be imperforate, then the connection is described, along with the presence of an imperforate valve. It is also necessary in hearts with single outlet to describe the ventricular origin of the arterial trunk. This may be exclusively from a right or a left ventricle, but more usually the trunk overrides the septum, taking its origin from both ventricles.

There are fewer morphologies for the valves at the ventriculo-arterial than at the atrioventricular junctions. A common arterial valve can only exist with a specific type of single outlet, namely common arterial trunk. Straddling of an arterial valve is impossible because it has no tension apparatus. Thus, the possible patterns are two perforate valves, one or both of which may override, or one perforate and one imperforate valve. As with overriding atrioventricular valves, the degree of override of an arterial valve determines the precise origin of the arterial trunk from the ventricular mass, the overriding valve, or valves, being assigned to the ventricle supporting the greater part of its circumference. When making this decision, as with atrioventricular connections, we err on the side of the more usually encountered pattern.

## ARTERIAL RELATIONSHIPS

Relationships are usually described at valvar level, and many systems for nomenclature have been constructed on this basis. It is our practise to describe arterial valvar relationships in terms of both right-left and anterior- posterior coordinates. In this way, aortic valvar position is described relative to the pulmonary trunk in terms of eight positions of a compass, using the simple terms left, right, anterior, posterior and side-by-side in their various combinations. As long as we then remember that these describe only arterial valvar relations, and convey no information about either the origin of the arterial trunks from the ventricular mass, or the morphology of the ventricular outflow tracts, we have no fear of producing confusion.

From the stance of positions of the arterial trunks, the possibilities are either for the pulmonary trunk to spiral round the aorta as it ascends from the base of the ventricles, or for the two trunks to ascend in parallel fashion. It is rarely necessary to describe these relationships. Spiralling trunks are associated most frequently with concordant ventriculo-arterial connections, and parallel trunks with discordant or double outlet connections, but again there is no predictive value in these relationships. In almost all hearts, the aortic arch crosses superiorly to the bifurcation of the pulmonary arteries. The side of the aortic arch depends on whether it passes to the right or left of the trachea. The position of the descending aorta is defined relative to the vertebral column.

## INFUNDIBULAR MORPHOLOGY

The infundibular regions are no more and no less than the outlet components of the ventricular mass. If the infundibular structures are recognized for what they are, and their morphology described as such, then they, too, provide no problems in recognition and description. The morphology of the ventricular outlet portions is variable for any heart. Potentially, each ventricle can possess a complete muscular funnel as its outlet portion, and then each arterial valve can be said to have a complete infundibulum. When considered as a whole, the outlet portions of the ventricular mass in the setting of bilateral infundibulums have three discrete parts. Two of the parts form the anterior and posterior halves of the funnels of muscle supporting the arterial valves. The anterior, parietal, part is the free anterior ventricular wall. The posterior part is the inner heart curvature, a structure that separates the leaflets of the arterial from those of the atrioventricular valves. We term this component the ventriculo-infundibular fold. The third part is the septum that separates the two subarterial outlets, which we designate the outlet, or infundibular, septum. It is possible, albeit rarely, for both arterial valves to be separated from both atrioventricular valves by the ventriculo-infundibular fold, but for the arterial valves to be in fibrous continuity with one another because of the absence of the outlet septum. In most hearts, however, some part of the infundibular musculature is effaced so that fibrous continuity occurs between the leaflets of one of the arterial and the atrioventricular valves. Most frequently, it is the morphologically left ventricular part of the ventriculo-infundibular fold that is attenuated. As a result, there is fibrous continuity between the leaflets of the mitral valve and the arterial valve supported by the left ventricle. Whether the arterial valve is aortic or pulmonary will depend on the ventriculo-arterial connections present. In the usual arrangement, the morphologically right ventricular part of the ventricular-infundibular fold persists, so that there is tricuspid-arterial valvar discontinuity. Depending on the integrity of the outlet septum, there is usually a completely muscular outflow tract, or infundibulum, in the morphologically right ventricle. When both outlet portions are connected to the morphologically right ventricle, then the ventriculo-infundibular fold can persist in its entirety, producing discontinuity bilaterally between the leaflets of the atrioventricular and arterial valves. But many hearts in which both arterial valves are connected unequivocally to the right ventricle have fibrous continuity between at least one arterial valve and an atrioventricular valve. It makes little sense to deny the presence of origin of both arterial trunks from the right ventricle in this setting. This situation is yet another example of the controversy generated when one feature of cardiac morphology is determined on the basis of a second, unrelated, feature. When both arterial trunks take their origin from the morphologically left ventricle, the tendency is for there to be continuity between the leaflets of both arterial valves and both atrioventricular valves. Even then, in some instances, the ventriculo-infundibular fold may persist in part or in its whole.

It is usually the state of the ventriculo-infundibular fold, therefore, that is the determining feature of infundibular morphology. Ignoring the rare situation of complete absence of the outlet septum, and considering morphology from the standpoint of the arterial valves, there are four possible arrangements. First, there may be a complete subpulmonary infundibulum, with continuity between the leaflets of the aortic and atrioventricular valves. Second, there may be a complete subaortic infundibulum, with continuity between the pulmonary and the atrioventricular valves. Third, there may be bilateral infundibulums, with absence of continuity between the leaflets of the arterial and atrioventricular valves. Fourth, there may be bilaterally deficient infundibulums, with continuity bilaterally between the arterial and the atrioventricular valves. In themselves, these terms are not specific. For specificity, it is also necessary to know which arterial valve takes origin from which ventricle. This emphasizes the fact that infundibular morphology is independent of the ventriculo-arterial connections.

## ASSOCIATED MALFORMATIONS

The majority of patients seen with congenitally malformed hearts will have their cardiac segments joined together in usual fashion, together with normal morphology and relations. In such a setting, the associated malformation will be the anomaly. It is also necessary, nonetheless, to pay attention to the position of the heart within the chest, and the orientation of the cardiac apex, recognising that the heart may be positioned ectopically outside the thoracic cavity. An abnormal position of the heart within the chest is another associated malformation, and should not be elevated to a prime diagnosis. This is not to decry the importance of an abnormal cardiac position, if only to aid in interpretation of the electrocardiogram. Knowing that the heart is malpositioned, however, gives no information concerning its internal architecture. Full sequential segmental analysis is needed to establish the cardiac structure, and not the other way round. The heart can be located mostly in the left hemithorax, mostly in the right hemithorax, or centrally positioned in the mediastinum. The cardiac apex can then point to the left, to the right, or to the middle. The orientation of the apex is independent of cardiac position. Both of these features are independent of the arrangement of the atrial appendages, and of the thoracic and abdominal organs. Describing a right-sided heart, with leftward apex, should be understandable by all, even including the patient, or his or her parents.

## CONCLUSIONS

The system we have described is simple, and accounts for all malformations, even if a combination of lesions has never previously be en encountered or described. It depends only on recognition of the anatomy as it is observed. With the newly available techniques of three-dimensional imaging, all the features described should be seen as readily by the clinician as by the morphologist. Thus, there is hope that polemics concerning description of congenitally malformed hearts will now be viewed as part of the history of evolution of our specialty.
